# Thank You to All Our Reviewers in 2022

**DOI:** 10.1055/s-0043-1764391

**Published:** 2023-03-24

The editorial board of Avicenna Journal of Medicine would like to thank the following reviewers who have reviewed manuscripts for the journal in 2022:

Rami Abazid
Aula Abbara
Kenneth Abbott
Yazan Abboud
Heba Abolaban
Fadee Abualrub
Manik Aggarwal
Ashfaque Ahmad
Rizwan Ahmed
Arif Al Mulla
Moamen Al Zoubi
Mousa Al-Abbadi
Hatim Al-Maghraby
Ghiath Alahamad
Fares Alahdab
Mohamad Alhoda Alahmad
Monirah Albathi
Muhamad Alhaj Moustafa
Eyad Almallouhi
Nidal M. Almasri
Houssam Alnahhas
Muhammad Alsayid
Osama Altayar
Mohammad Altujjar
Nabil Alzaiem
Haitham Arabi
Ziad Arabi
Mustafa Arslan
Hrayr Attarian
Heyam Awad
Bilal Azab
Muhammad Eyad BaAth
Mohamad Badawi
Magdy Hasan Balaha
Walid Ballourah
Josh Bernstein
Mustafa Boğan
Abdelkader Chaar
Arjun Chatterjee
Basem Dahshan
Anju Dinkar
Mainak Dutta
Moutaz El Kadri
Ibrahim H. Elkhidir
Abdel-Naser Y. Elzouki
Arife Erdoğan
Sana Farooki
Hee Kong Fong
Khaled Ghanem
Varun Goel
Umut Gulacti
Tarik Hadid
Ali Hajeer
Abdulrahman Hallak
Mohammad Khalid Hamza
Ibrahem Hanafi
Bashar Hasan
Mohammad Helwani
Arda Isik
Meenu Jain
Hasan Jamil
Andrew Juergens
Ahmad Kaako
Mohammed Kabbesh
Nashwa Nabil Kamal
Yassar Kanawati
Anas Kawayeh
Meghit Boumediene Khaled
Ahsan Khan
Sarita Khare
Said Khayyata
Omar Khorfan
Brenda Klement
Ahmad Mansour
Murlean Mills
Abdul Mohammed
Emir Muzurović
Antoine Aysar Naem
Hassan Nagy
Alhawiti Naif
Hala Nas
Mahmoud Nassar
Tarek Nayfeh
Ahmed Abd Al-Wahab Nugud
Waseem Ostwani
Serdar Özdemir
Seyedmahdi Pahlavani
Gauri Pandit
Neethi Paranji
Boonchu Pattama
Mona Kyndi Pedersen
Magdalena Pertynska-Marczewska
Rayan Jo Rachwan
Fouad Sharif Sharif Sabatin
Hossein Salimnia
Malik Sallam
Tamara Salloum
Abdulghani Sankari
Ibrahim M Sayed
Parvez Shaikh
Mohammed Nader Shalaby
Ardalan Shariat
Aryan Shiari
Saurabh RamBihariLal Shrivastava
Mohamad Bassam Sonbol
Murgesan Subramaniam
Ammar Sukari
Jaya Thilakan
Ben Tucker
Ufuk Uylas
Karthikeyan Venkatachalam
Vijaya Krishna Prasad Vudathaneni
Naveen Yarlagadda
Erdal Yavuz
Weng-Liang Yu
Obada Zayegh
Yahia Zeino

